# Supplementary material for: Complexome profiling of the Chlamydomonas psb28 mutant reveals TEF5 as an early PSII assembly factor
Source: Plant Cell. 2025 Mar 17;37(6):koaf055. doi: 10.1093/plcell/koaf055 (PMC12164590; doi:10.1093/plcell/koaf055)
Supplement: koaf055_Supplementary_Data [file koaf055_supplementary_data.zip › psb28_interactive.zip!/plots/intensity/qualityPlots/peptides/_Cre04.g217933.t1.1.html]

### Cre04.g217933.t1.1

Trivial name:   
SeqCoverage [median]: 11.20  
Mass: 37.19 kDa  
  
 

| Sequence | MissedCleavage | Mass [Da] | wtIntensity | muIntensity | Score |
| --- | --- | --- | --- | --- | --- |
| AGLASLVGQR | 0 | 970.56 | 385.10 | 2598.56 | 73.26 |
| ASEVTLGGSIR | 0 | 1088.58 | 0.00 | 1006.72 | 88.50 |
| LLVAQTQR | 0 | 927.55 | 342.93 | 0.00 | 67.03 |
| YGVTGGGQDLR | 0 | 1121.55 | 0.00 | 425.70 | 71.45 |
|  |  |  | 728.0 | 4031.0 |  |
